# Certain Drugs on the Circulation of the Kidneys

**Published:** 1887-10

**Authors:** Charles D. F. Phillips

**Affiliations:** London


### *THE ACTION OF CERTAIN DRUGS ON THE CIRCULATION AND SECRETION OF THE KIDNEY.*

By Dr. CHARLES D. F. PHILLIPS, of London.

This contained a number of very interesting experiments, made with Roy's onkometer, with caffein, spartein, strophanthin, digitalin and ulexin[1]. He concluded that the flow of urine is not so much dependent on the blood-pressure as on the rate of flow of the blood in the renal vessels. With regard to this point, it is necessary to remember that, although such drugs as strophanthin,

(1) Ulexin is an alkaloid from the gorse *ulexis europæus*.

producing a great increase in the *force* of the cardiac beats, yet these are very much slowed, so that it is quite possible that although the heart's action is stronger, yet the total amount of blood sent through any given organ, such as the kidney, in a given time, may remain the same. Whereas, such a drug as digitalis, producing, as it does, a rise of blood-pressure and a contraction of the kidney vessels, may cause an increased quantity of blood to pass through the renal vessels. On this view, one could find the explanation of digitalin being a diuretic, and strophanthin not being one.

Inasmuch, however, as spartein has not so marked diuretic action, we must also assume that digitalin must have some peripheral action on the secretory apparatus of the kidney.

His results were tabulated briefly as follows, in three divisions:

(A) DRUGS THAT FIRST CONTRACT AND AFTERWARD DILATE THE KIDNEY.

(1) *Caffein*—in small doses—induces in the stage of contraction a fall of blood-pressure, in that of expansion, a slight rise; during the former, the flow of *urine* may be arrested; during the latter it is always increased, such increase depending on dilatation of renal vessels.

(The possible *arrest* of secretion during the first stage is special to caffein, and may be induced by large or repeated doses.)

(2) *Ulexin*—1/6 gr. greatly raises blood-pressure during the first stage (that of contraction); in the second, expansion is much greater in degree, but shorter in duration, than under caffein, and is accompanied by brief but marked increase in urinary flow; the effective dose is limited by its toxic action on respiratory centres. Practically, *excess* of caffein induces only the first stage; *excess* of ulexin only the second.

(B) SUBSTANCES THAT DILATE THE KIDNEY, BUT TO LESS EXTENT AND MORE SLOWLY THAN CAFFEIN AND ULEXIN, are dextrose, urea, sodium, chloride and acetate, and probably all constituents of the urine.

(C) DRUGS THAT CONTRACT THE KIDNEY WITHOUT SUBSEQUENT EXPANSION.

(1) Digitalin, with *increased* secretion of urine (probably resulting from general heightened blood-pressure).

(2) Spartein, with *diminished* secretion (in health, at least).

(3) Strophanthin causes slight temporary contraction, with no marked increase of secretion.

(4) Apocynëin, similar temporary contraction, and no definite increase of secretion.

(5) Turpentine; (6) adonidin; and (7) barium chloride give similar results.

In conclusion, it seemed to him that the plethysmographic method of experimentation is a valuable one for determining the exact action of drugs on the circulation, and one that deserves more attention than it has hitherto attracted.

Dr. Murrell referred to the importance of this study of diuretics, since so little is known, comparatively, of their effects. Diaphoretics are well understood, but the action of diuretics remain to be worked out. He could not understand the points of superiority of the instrument used over Marcy's tympanum.

